# British Laryngological, Rhinological, and Otological Association

**Published:** 1898-11-19

**Authors:** 


### BRITISH LARYNGOLOGICAL, RHINOLOGICAL, AND OTOLOGICAL ASSOCIATION.

At the meeting held on October 28th, the President (J. Middlemas Hunt, M.B., C.M.) exhibited a case of epithelioma of larynx with microscopical specimen in a man, aged 44, who had suffered from increasing hoarseness for six months, and was now quite aphonic. Dr. Hunt proposed to perform thyrotomy, and remove the growth and surrounding parts. Mr. Mayo Collier advocated removal of half the larynx. Mr. Bark (Liverpool) spoke in favour of thyrotomy as preferable to removal of half the larynx in cases like the one under discussion. If, after splitting the thyroid, the growth was found to be more extensive than was apparent by laryngoscopic examination half the larynx might then be removed. Dr. Dundas Grant advocated exploratory thyrotomy. If more than this were required, entire removal would be necessary, as the growth affected both sides of the larynx. Mr. Lennox Browne expressed his opinion in favour of thyrotomy. Dr. Furness Potter showed a case of loss of abductor movement in left vocal cord. The patient (a milkman) a healthy-looking man, aged 33 years, had been under treatment during the last two years for chronic pharyngitis and nasal polypi. There was complete absence of any sign or symptom to indicate the cause of loss of motor power. Dr. Whistler suggested that pressure on the recurrent might be caused by enlargement of one of the chain of laryngo-tracheal glands, which being so deeply situated would be most difficult, if not impossible, to detect by palpation. He recalled a case of his own where this diagnosis had been verified by post-mortem examination. Mr. Mayo Collier cited a case in which paralysis of a vocal cord was caused by an empyema situated over the apex of the lung, and in which motor power returned after surgical treatment of the empyema. Dr. Milligan (Manchester) thought a number of cases of paralysis of the left cord were due to the presence of enlarged intrathoracic glands, which could not be detected during life. Dr. Dundas Grant referred to the more recently discovered causes, such as syringomyelia and alcoholic neuritis, but thought there would always remain a certain percentage of cases in which the cause was undiscoverable. Dr. Milligan read the notes of a case of suppurative catarrh of the right maxillary antrum and right frontal sinus, in which, at the time of operation on the antrum, though a careful examination was made, frontal sinus suppuration was not diagnosed. Two months later the onset of neuralgic pain in the neighbourhood of the right frontal sinus made it probable that it was the site of suppurative inflammation. On opening the sinus from the outside it was found to be full of pus. Dr. Milligan was of opinion that the frontal sinus trouble had developed subsequently, and was due to infection from the antrum (possibly pus having been driven up during the manipulations necessary for cleansing the antrum). The President delivered his inaugural address on "An Appreciation of Three British Aurists—Wilde, Toynbee, and Hinton," dwelling strongly on the respect in which these workers were held by Continental authorities on aural surgery. Various other cases were shown, and Dr. Milligan described two cases of extradural abscess following chronic suppurative middle-ear disease.

